# Frailty and *delirium* in hospitalized older adults: A systematic review with meta-analysis

**DOI:** 10.1590/1518-8345.6120.3687

**Published:** 2022-10-17

**Authors:** Clovis Cechinel, Maria Helena Lenardt, João Alberto Martins Rodrigues, Maria Angélica Binotto, Márcia Marrocos Aristides, Rosane Kraus

**Affiliations:** 1 Universidade Federal do Paraná, Curitiba, PR, Brazil.; 2 Secretaria Municipal de Saúde, Hospital Municipal do Idoso Zilda Arns, Curitiba, PR, Brazil.; 3 Universidade Estadual do Centro-Oeste, Departamento de Educação Fisica, Irati, PR, Brazil.; 4 Fundação Estatal de Atencão a Saúde, Hospital Municipal do Idoso Zilda Arns, Curitiba, PR, Brazil.

**Keywords:** Systematic Review, Meta-Analysis, Hospitalization, Frail Elderly, Delirium, Prevalence, Revisão Sistemática, Metanálise, Hospitalização, Idoso Fragilizado, Delirium, Prevalência, Revisión Sistemática, Metanálisis, Hospitalización, Adulto Mayor Frágil, Delirium, Prevalencia

## Abstract

**Objective:**

to estimate the prevalence and synthesize diverse evidence about the relationship between frailty and *delirium* in hospitalized older adults.

**Method:**

a systematic review with meta-analysis in which observational studies conducted with older adults about frailty, *delirium* and hospitalization, were selected without time of language restrictions. The search was conducted in the MEDLINE, EMBASE, CINAHL, Scopus, Web of Science and CENTRAL databases during August 2021. The precepts set forth by the Joanna Briggs Institute (JBI) - Evidence Synthesis Groups were followed. The meta-analysis model estimated the relative risk corresponding to the prevalence of frailty and *delirium.* The inverse variance method for proportions was used to estimate the prevalence values and relative risks for binary outcomes.

**Results:**

initially, 1,244 articles were identified, of which 26 were included in the meta-analysis (n=13,502 participants), with 34% prevalence of frailty (95% CI:0.26-0.42; *I* ^2^=99%; *t* ^2^=0.7618, *p*=0) and 21% for *delirium* (95% CI:0.17-0,25; *I* ^2^=95%; *t* ^2^=0.3454, p<0.01). The risk for hospitalized older adults to develop *delirium* was 66% (RR: 1.66; 95% CI:1.23-2.22; I^2^=92%; t^2^=0.4154; *p*<0.01).

**Conclusion:**

34% prevalence of frailty and 21% of *delirium* in hospitalized older adults, with frailty being an independent risk factor for developing *delirium*, with an increased chance of 66% when compared to non-frail individuals.

## Resumo

**Objetivo:**

estimar a prevalência e sintetizar evidências sobre a relação entre fragilidade e *delirium* em idosos hospitalizados.

**Método:**

revisão sistemática com metanálise na qual foram selecionados estudos observacionais realizados com idosos sobre fragilidade, *delirium* e hospitalização, sem recortes temporais e de idioma. A busca foi realizada nas bases de dados MEDLINE, EMBASE, CINAHL, Scopus, Web of Science e CENTRAL durante o mês de agosto de 2021. Foram seguidos os preceitos estabelecidos pelo *Joanna Briggs Institute* (JBI) - Grupos de Síntese de Evidências.*.* O modelo de metanálise estimou risco relativo da prevalência de fragilidade e *delirium.* Utilizou-se método da variância inversa para proporções para estimar as prevalências e risco relativo para desfechos binários.

**Resultados:**

identificaram-se, inicialmente, 1.244 artigos, 26 incluídos na metanálise (n=13.502 participantes), sendo a prevalência de fragilidade 34% (IC 95% 0,26 a 0,42; *I* ^2^=99%; *t* ^2^= 0,7618, *p*=0) e *delirium* 21% (IC 95% 0,17 a 0,25; *I* ^2^=95%; *t* ^2^= 0,3454, p<0,01). O risco do idoso frágil hospitalizado desenvolver *delirium* foi de 66% (RR 1,66; IC 95% 1,23 a 2,22; I^2^=92%; t^2^=0,4154; *p*<0,01).

**Conclusão:**

prevalência de 34% de fragilidade e 21% de *delirium* em idosos hospitalizados, sendo a fragilidade um fator de risco independente para desenvolvimento de *delirium*, com um aumento de chance de 66% comparado aos não frágeis.

## Resumen

**Objetivo:**

estimar la prevalencia y sintetizar evidencias sobre la relación entre fragilidad y *delirium* en adultos mayores hospitalizados.

**Método:**

revisión sistemática con metanálisis en el que se seleccionaron estudios observacionales realizados con adultos mayores sobre fragilidad, *delirium* y hospitalización, sin recorte temporal ni de idioma. La búsqueda se realizó en las bases de datos MEDLINE, EMBASE, CINAHL, *Scopus*, *Web of Science* y CENTRAL en agosto de 2021. Se siguieron los preceptos del Instituto Joanna Briggs (*Joanna Briggs Institute, JBI) - Evidence Synthesis Groups*. El modelo de metanálisis estimó el riesgo relativo de la prevalencia de fragilidad y *delirium*. Se utilizó el método de la varianza inversa para proporciones para estimar la prevalencia y el riesgo relativo de los desenlaces binarios.

**Resultados:**

inicialmente se identificaron 1.244 artículos, se incluyen en el metanálisis 26 (n=13.502 participantes), la prevalencia de fragilidad fue del 34% (IC 95% 0,26 a 0,42; *I* ^2^=99%; *t* ^2^= 0,7618, *p*=0) y de *delirium* del 21% (IC 95% 0,17 a 0,25; *I* ^2^=95%; *t* ^2^= 0,3454, p<0,01). El riesgo de que el adulto mayor frágil hospitalizado desarrolle *delirium* fue del 66% (RR 1,66; IC 95% 1,23 a 2,22; I^2^=92%; t^2^=0,4154; *p*<0,01).

**Conclusión:**

los adultos mayores hospitalizados tienen una prevalencia de fragilidad del 34% y de *delirium* del 21%, la fragilidad es un factor de riesgo independiente para el desarrollo de *delirium*, cuando se compara a los frágiles con los no frágiles, la probabilidad de *delirium* de los primeros es de un 66% más.

**Highlights**

(1) The prevalence of frailty in hospitalized older adults was 34% (from 23% to 46%).

(2) The prevalence of *delirium* in hospitalized older adults was 21% (from 17% to 24%).

(3) The relative risk of frailty and delirium was 1.66% (from 1.18% to 2.33%, p<0.001).

(4) Frailty is an independent risk factor for developing *delirium*.

(5) Frailty can be a therapeutic target in the prevention of *delirium*.

## Introduction

Older adults constitute a unique population segment in hospital care, and the assistance team must be aware of the particularities of this age group, especially the syndromes, in order to detect and treat them early in time^1^. Physical frailty deserves to be highlighted among such conditions due to its multicausal nature, being defined as follows: “a clinical condition characterized by increased vulnerability in the individual when exposed to internal and external stressors, and is a major contributor to functional decline and early mortality in older adults”^2^. 

In the scoping review, 204 studies were evaluated on the theme of frail older adults hospitalized with acute diseases, 14% from the geriatrics and emergency areas and 11% from the general practice. Of the 204 studies, 67% identified frail participants using the “Frailty Phenotype”, the “Clinical Frailty Scale” (CFS) and the “Frailty Index” (12% each). In this review, 74% of the studies showed a correlation between frailty and the “morality” and “hospitalization time” outcomes^3^.

*Delirium* is another condition that affects the hospitalized aged population. It is a form of acute brain dysfunction^4^ characterized by a sudden change in the level of attention and by an altered level of consciousness that fluctuates over time. The American Psychiatric Association’s definition, according to the Diagnostic and Statistical Manual of Mental Disorders, Fifth Edition, is “a mental disorder of acute onset and fluctuating course characterized by disturbances of consciousness, orientation, memory, thinking, perception and behavior”^5^. It is associated with a decrease in functional status, to institutionalization, to premature mortality, and to an increase in health-related costs. 

One-third of the clinical inpatients aged at least 70 years old have *delirium*; the condition is present in half of these patients on admission and develops during hospitalization in the other half^6^. Its etiology is multifactorial, with an incidence rate of 83% in hospitalized older adults^5^. 

Frailty and *delírium* share responsibility for the increase in morbidity and mortality^7^, in addition to prolonged hospitalization times and long-term functional and cognitive impairment^8^. In an emergency service, it was verified that *delirium* had 3 time more chances to occur in frail than in non-frail older adults after adjustments for age and gender^9^. 

A prospective cohort study conducted in Italy with 89 hospitalized older adults evaluated *delirium*, attention performance and frailty status in a geriatric emergency department. To evaluate the patients’ attention, they were asked to list the months of the year backwards (MOTYB test), then list the days of the week backwards (DOWB) and count from 20 to 1 (BC). The mean age was 83.1 ± 6 years old and prevalence values of 47.19% (n=42) and 41.70% (n=37) were observed for frailty and *delirium*, respectively. There was an association between frailty and *delirium* (RR: 4.90; 95% CI:2.01-11.94)^10^.

The association of the frailty level on admission to the emergency service with hospital complications, including *delirium*, was evaluated in the emergency room of two public general hospitals in Mexico City - Mexico. This secondary analysis of the cohort study conducted with 548 individuals presented a mean age of 76 ± 7.2 years old. The presence of *delirium* according to frailty stratification was 0% (frailty index <0.2), 3.4% (frailty index from 0.20 to 0.39), 6.2% (frailty index from 0.40 to 0.59) and 23.2% (frailty index >0.60); thus, frailty was positively associated with *delirium* (β = 3.68; 95% CI: 1.53-5.83, *p*<0.01)^11^. 

The literature regarding *delirium* and frailty in hospitalized older adults is scarce, being mainly limited to the mortality outcome or to specific subgroups such as hospital sectors or related to surgical procedures.

Relevance of the topic is noted due to the fact that physical frailty and *delirium* proved to be two of the most complex management problems among hospitalized older adults. In the clinical practice, occurrence of these conditions is constantly observed among hospitalized older adults, and they are related to negative outcomes such as delayed functional recovery, disability^12^ and death^13^.

Given the above, the objective of the current study was to estimate the prevalence and synthesize diverse evidence about the relationship between frailty and *delirium* in hospitalized older adults.

## Method

This is a systematic review with meta-analysis, based on the precepts set forth by the Joanna Briggs Institute (JBI) - Evidence Synthesis Groups^14^. The “Association of delirium and fragility in hospitalized elderly: systematic review” protocol is published on the International Platform of Registered Systematic Review and Meta-analysis Protocols (INPLASY), DOI: 10.37766/inplasy2021.9.0022. 

### Research strategy

In order to formulate the guiding question and design the search for studies, the PEO (P - Population or Patients; E - Exposure; O - Outcomes)^15^ was used, where P (Frail older adults), E (Hospitalization) and O (*Delirium*). After applying the PEO strategy, and to guide the search strategy terms, the following question was formulated: Which is the relationship between frailty and *delirium* in hospitalized older adults? 

The inclusion criteria to select the study were as follows: observational studies, including prospective and retrospective cohort, case-control and cross-sectional studies; presence of the variables of interest: “frailty” and “*delirium”*; developed in a hospital setting; involving older adults aged ≥ 60 years old; and published in any language with no limitation regarding publication date. The exclusion criteria for the studies were as follows: not categorizing patients as frail and non-frail, case reports, letters to the editor, abstracts in conference proceedings, dissertations, theses and monographs.

### Search and selection of the studies

The search strategy was specific to each database and initially used the Medical Subject Headings (MeSH) descriptors, later translated into specific descriptors (*Descritores em Ciências da Saúde*, DeCS) and Embase Subject Headings (Emtree). The search strategy was applied by the main researcher in the MEDLINE (PubMed Portal); SciELO*;* BVS*;* EMBASE, CINAHL, Scopus, Web of Science (CAPES Journals Portal) and CENTRAL (Cochrane) databases in August 2021; the following search terms (MeSH): Aged, Frailty, Frail Elderly, Inpatients, *Delirium*, Hospitalization and the free terms: Elderly, Frailties, Frailness, Frailty Syndrome, Debilities, Functionally Impaired Elderly, Frail Older Adults, Subacute *Delirium*, Mixed Origin *Delirium* were associated by means of the Boolean operators (OR and AND) and structured the specific search strategy for each database, as described in the systematic review protocol registration. 

### Data extraction and synthesis

The total number of articles found in each database and the sum of all databases were recorded in the PRISMA flowchart^16^, as well as the entire selection process and reasons for exclusion. The results of the searches were imported into the Mendeley^®^ software to store, organize and classify the references. In addition to that, it was possible to remove the duplicates in the reference manager.

The database search was performed by the main researcher, who then divided the titles of the articles between two reviewers who performed the evaluation independently.

The titles of the articles were analyzed and the ineligible studies were excluded. In the subsequent stage, the abstracts were read and the ineligible articles were removed after applying the eligibility criteria. 

The abstracts evaluated were returned to the main researcher, who made all articles available in full-text format to the reviewers for evaluation of the eligibility criteria. To minimize a possible bias in selection of the studies, a refinement procedure was performed by two independent reviewers seeking 100% agreement, and a third reviewer evaluated the possible divergences that occurred in the selection of abstracts to make a final decision on their inclusion or exclusion.

Data extraction was performed in a Microsoft Excel^®^ table to compile the data from the studies included. It was constructed to cover the previously defined eligibility criteria using the Joanna Briggs Institute^14^ instruments, which included the following: author’s name, year, country, patient’s profile, purpose of the paper, sample size, study design, frailty evaluation instrument, *delirium* evaluation instrument and outcomes. The final references of the primary studies included were also evaluated manually, in an attempt to find relevant articles that might be added to the review. 

To describe the intensity of agreement between the reviewers, the Kappa measure was used, which is based on the number of concordant answers, i.e., the frequency at which the result is the same between the reviewers^17^. For this study, the Kappa agreement index was 0.892, which shows strong/almost perfect agreement between the reviewers.

The data analyzed for the meta-analysis were the following: total number of patients, number of frail and non-frail patients, number of patients with *delirium* and their combined effects. The meta-analysis model estimated the relative risk corresponding to the prevalence of frailty and *delirium.* The “pooled effects” were estimated using the inverse variance method of proportions to estimate prevalence values and relative risk for the binary outcomes, with 95% confidence interval, and represented in Forest plots. 

Heterogeneity across the studies was tested by means of the I^2^ test, considering it significant when *p*<0.05. The alternative hypothesis of the heterogeneity test is that variability/heterogeneity is significant; therefore, fixed or random effects models were chosen based on acceptance or rejection of the null hypothesis. All the analyses were performed in the R 4.1.1 environment^18^.

### Evaluation of the methodological quality

The eligible studies were critically evaluated by two independent reviewers regarding their methodological quality by resorting to the Joanna Briggs Institute (JBI) scale. Any and all disagreements were solved by means of a discussion with a third reviewer. On a scale consisting of nine criteria, studies that met from zero to three criteria were considered to be of low quality, those that met from four to six criteria were considered to be of medium quality, and from seven or more were considered to be of high methodological quality. The evaluation scores in relation to the methodological quality showed that most of the articles are of average to high quality. Regardless of the results referring to their methodological quality, all the articles were submitted to data extraction and synthesis, as can be seen in Figure 1.

**Figure 1:** Result of the methodological evaluations of the articles included in the study. Curitiba, PR, Brazil, 2021

AUTHOR (DATA)	1. Is the sample clipping appropriate to adress the target population?	2. Were the study participants properly sampled?	3. Was the sample size adequate?	4. Were the study participantes and design described in detail?	5.Was data analysis performed with sufficient sample coverage?	6. Were valid methods used to identify the condition?	7. Was the condition assessed in a standardized and reliable way?	8. Was na appropriate statistical analysis performed?	9. Was the response rate adequate and, if not, was the low response rate properly managed?	Total
Leung; Tsai; Sands, 2011 ^17^	(-)	(+)	(-)	(+)	(+)	(+)	(+)	(+)	(+)	7
Eeles et al., 2012 ^18^	(+)	(+)	(-)	(-)	(+)	(+)	(+)	(-)	(+)	6
Joosten et al., 2014 ^19^	(+)	(I)	(+)	(+)	(+)	(+)	(+)	(+)	(+)	8
Hempenius et al., 2014 ^20^	(-)	(+)	(+)	(+)	(+)	(+)	(U)	(+)	(+)	7
Eide et al., 2015 ^21^	(+)	(+)	(-)	(+)	(+)	(+)	(U)	(+)	(+)	7
Partridge et al., 2015 ^22^	(+)	(+)	(+)	(+)	(+)	(+)	(U)	(+)	(+)	8
Nguyen; Cumming; Hilmer, 2016 ^23^	(-)	(+)	(+)	(+)	(+)	(+)	(U)	(+)	(+)	7
Assmann et al., 2016 ^24^	(-)	(+)	(-)	(+)	(+)	(+)	(+)	(+)	(+)	7
Chew et al., 2017 ^25^	(+)	(+)	(+)	(+)	(+)	(+)	(+)	(+)	(+)	9
Ogawa et al., 2017 ^26^	(+)	(+)	(+)	(+)	(+)	(-)	(-)	(+)	(+)	7
Gleason et al., 2017 ^27^	(-)	(+)	(+)	(+)	(+)	(+)	(-)	(+)	(+)	7
Dani et al., 2018 ^28^	(+)	(+)	(+)	(+)	(+)	(+)	(+)	(+)	(+)	9
Tanaka et al., 2018 ^29^	(+)	(+)	(+)	(+)	(+)	(+)	(U)	(+)	(+)	8
Giroux et al., 2018 ^9^	(+)	(+)	(-)	(+)	(+)	(+)	(+)	(+)	(-)	7
Alabaf Sabbaghi et al., 2018 ^30^	(+)	(+)	(+)	(+)	(+)	(-)	(U)	(+)	(-)	6
Nomura et al., 2018 ^31^	(-)	(+)	(-)	(+)	(+)	(+)	(U)	(+)	(+)	6
Goudzwaard et al., 2018 ^32^	(-)	(+)	(-)	(+)	(+)	(+)	(U)	(+)	(+)	6
Geriatric Medicine Research Collaborative, 2019 ^33^	(+)	(+)	(+)	(-)	(+)	(+)	(+)	(+)	(+)	8
Saravana-Bawan et al., 2019 ^34^	(+)	(+)	(+)	(+)	(+)	(+)	(U)	(+)	(+)	8
Bellelli et al., 2019 ^10^	(-)	(+)	(-)	(+)	(+)	(+)	(U)	(+)	(+)	6
Goudzwaard et al., 2020 ^35^	(+)	(+)	(+)	(+)	(+)	(+)	(+)	(+)	(+)	9
Itagaki et al., 2020 ^36^	(+)	(+)	(-)	(+)	(+)	(+)	(U)	(+)	(-)	6
Saljuqi et al., 2020 ^37^	(+)	(+)	(+)	(-)	(+)	(+)	(U)	(+)	(+)	7
Susano et al., 2020 ^38^	(+)	(+)	(+)	(+)	(+)	(+)	(-)	(+)	(+)	8
Mahanna-Gabrielli et al., 2020 ^39^	(+)	(+)	(+)	(+)	(+)	(+)	(U)	(+)	(+)	8
Chen; Qin (2020) ^40^	(+)	(+)	(+)	(-)	(+)	(+)	(-)	(+)	(+)	7

Note: (-) Did not meet this criterion; (+) Met this criterion, (U) Uncertain/Not clear. The score varies between 0 and 9 and, the higher it is, the better the quality of the study.

### Ethical aspects

As this study resorted to articles from databases and did not involve human beings, it waived approval by the Research Ethics Committee, according to National Health Council Resolution No. 510/2016 and the ethical regulations in force^42^. 

## Results

**Figure 2:**
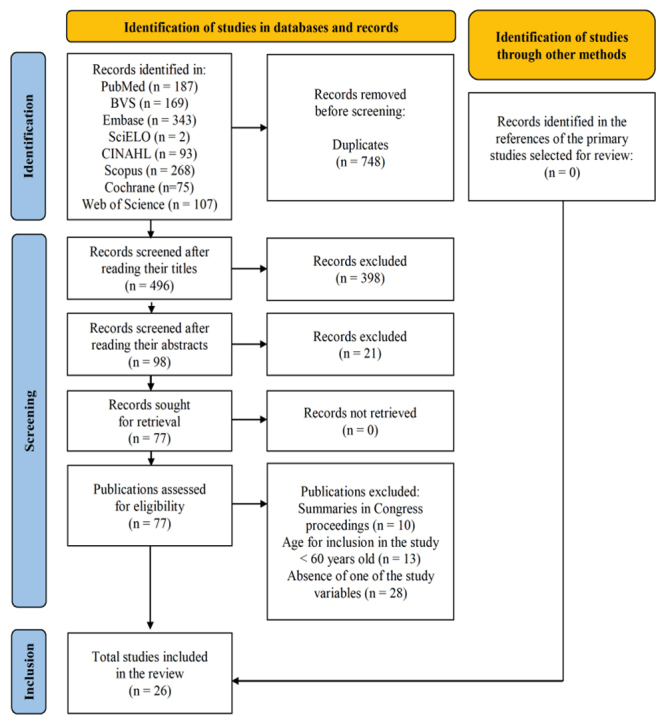
PRISMA flowchart corresponding to selection of the studies. Curitiba, PR, Brazil, 2021

The database search resulted in 1,244 studies in all eight databases; 748 were excluded for being duplicates and 496 were selected for reading their titles and abstracts. Of these, 398 articles were excluded after reading their titles and 21 after reading the abstracts, resulting in the selection of 77 for full-reading. A total of 51 were excluded after this stage, resulting in the inclusion of 26 articles. No new eligible studies for the review were found after consulting the references of the primary studies. Figure 2 shows the flowchart of the Preferred Reporting Items for Systematic Reviews and Meta-Analysis (PRISMA) method used to illustrate selection of the articles for this systematic review^16^. 

The reviewers included all the studies that met the inclusion and exclusion criteria, with discussion of the methodological weaknesses. A total of 13,502 participants (minimum of 63, maximum of 6,191) were included in the meta-analysis, with predominance of publications in 2019 and 2020 (n=5; 19.25%); 2018 (n=4; 15.38%); 2015 and 2017 (n=3; 11.55%); 2016 (n=2; 7.70%); and in 2011, 2012, 2014 and 2021 (n=1; 3.85%). 

The following stand out among the countries where the studies were conducted: United States of America (n=5; 19.,23%), followed by Japan (n=4; 15.38%), the Netherlands (n=4; 15.38%), United Kingdom (n=4; 15.38%), Canada (n=2; 7.69%), Australia (n=2; 7.69%), Singapore (n= 1; 3.84%), Norway (n=1; 3.84%), Italy (n=1; 3.84%), China (n=1; 3.84%) and Belgium (n=1; 3.84%). 

The most commonly employed frailty evaluation instrument was the Frailty Index (n=5; 19.23%); followed by the Clinical Frailty Scale (CFS) (n=4; 15.38%); the Frail Scale (n=3; 11.53%), the Erasmus Frailty Score (EFS), the Edmonton Frail Scale (n=2; 7.69%), the Japanese CHS version (j-CHS), the Kihon Checklist, the Frailty Index associated with clinical judgment; Groningen Frailty Indicator; Handgrip strength and gait speed; Erasmus Frailty Score, the Cardiovascular Health Study (CHS), Fried’s phenotype and modified Fried’s criteria (n=1; 3.84%). 

The most commonly used instrument for evaluation of *delirium* was the Confusion Assessment Method (CAM) (n=8; 30.75%); followed by the Intensive Care Delirium Screening Checklist (ICDSC) (n=3; 11.54%); uninformed (n=3; 11.54%); geriatric clinical evaluation (n=2; 7.70%); Diagnostic and Statistical Manual of Mental Disorders fifth edition (DSM-5) (n=2; 7.70%); Abbreviated Mental Test - 4 (4-ATM)/DSM-5 (n=2; 7.70%) and severity evaluation of the Delirium Rating Scale-Revised-98 (DRS-R-98), 4-ATM score, Confusion Assessment Method for the Intensive Care Unit (CAM-ICU), CAM/CAM-ICU, CAM/DSM-5, DSM-4 and (n=1; 3.35%). 

Figure 3 shows the distribution of the characteristics of the studies that comprised the *corpus* of the systematic review, with the following variables: author’s name, year, country, patient’s profile, objective of the paper, sample size, study design, frailty evaluation instrument, *delirium* evaluation instrument and outcomes.

**Figure 3:** Distribution of the characteristics of the studies that comprised the *corpus* of the systematic review. Curitiba, PR, Brazil, 2021

Authors	Origin	Type of patients	Objective	Sample size	Type of study	Age	Instrument for frailty	**Instrument for *delirium***	Results (95% CI^*^)
Leung; Tsai; Sands, 2011^19^	USA	Surgical, non-cardiac.	To investigate if preoperative frailty in surgical non-cardiac patients favors onset of postoperative *delirium*.	63	Prospective cohort study	>65 years old	Modified Fried Criteria	CAM^†^	Prevalence of frailty HR^‡^ 0.33 (0.23 - 0.45) Prevalence of *delirium* HR^‡^ 0.25 (0.16 - 0.37) Risk of *delirium* in frail patients RR^§^ 2.57 (1.11 - 5.94)
Eeles, et al., 2012^20^	Australia	Acute care, general hospital	To explore the relationship between *delirium* and frailty in aged patients and to determine their impact on survival.	273	Prospective cohort study	82.3±7.5	FI^||^ (33 items) (Frail>0.25)	DSM-5^¶^	Prevalence of frailty HR^‡^ 0.41 (0.35 - 0.47) Prevalence of *delirium* HR^‡^ 0.37 (0.31 - 0.43) Risk of *delirium* in frail patients RR^§^ 3.62 (2.54 - 5.18)
Joosten, et al., 2014^21^	Belgium	Geriatric ward	To evaluate the prevalence of frailty and to determine to which extent it predicts *delirium*, falls and mortality in hospitalized aged patients.	220	Prospective cohort study	CHS^**^: Frail 83.3±5.4 SOF^††^: 83.1±5.2	CHS^**^ and SOF^††^	CAM^†^	Prevalence of frailty HR^‡^ 0.40 (0.34 - 0.47) Prevalence of *delirium* HR^‡^ 0.11 (0.08 - 0.16) Risk of *delirium* in frail patients RR^§^ 1.07 (0.50 - 2.30)
Hempenius, et al., 2014^22^	Netherlands	Surgery, solid tumors	To determine the risk factors for postoperative *delirium* (POD) in aged patients with cancer.	251	Multicenter and retrospective cohort study	74.2±6.4 (65 - 92)	Groningen Frailty Indicator	CAM^†^	Prevalence of frailty HR^‡^ 0.34 (0.23 - 0.34) Prevalence of *delirium* HR^‡^ 0.18 (0.14 - 0.24) Risk of *delirium* in frail patients RR^§^ 2.01 (1.20 - 2.37)
Eide, et al., 2015^23^	Norway	Cardiac Surgery	To determine the incidence of postoperative *delirium* in people aged 80-89 years old subjected to TAVI^‡‡^ or to aortic valve surgical replacement; to identify risk factors and describe possible differences at onset and progression of postoperative *delirium* between the groups.	143	Prospective cohort study	> 80 83.5±2.7	SOF^††^	CAM^†^	Prevalence of frailty HR^‡^ 0.39 (0.31 - 0.47) Prevalence of *delirium* HR^‡^ 0.42 (0.34 - 0.50) Risk of *delirium* in frail patients RR^§^ 1.14 (0.85 - 1.53)
Partridge, et al., 2015^24^	United Kingdom	Elective and emergency arterial-vascular surgeries	To evaluate the prevalence of frailty, clinical conditions and functional status in preoperative among older adults undergoing arterial vascular surgery and to evaluate postoperative outcomes.	125	Prospective cohort study	76.3±7.27	EFS^§§^ (Frail≥6.5)	Not reported	Prevalence of frailty HR^‡^ 0.52 (0.43 - 0.61) Prevalence of *delirium* HR^‡^ 0.15 (0.10 - 0.22) Risk of *delirium* in frail patients RR^§^ 0.27 (0.17 - 0.43)
Nguyen; Cumming; Hilmer, 2016^25^	Australia	Clinical	To investigate the impact of frailty on mortality, hospitalization time and readmission in hospitalized aged patients with arterial fibrillation.	302	Prospective cohort study	84.7±7.1	EFS^§§^	Not reported	Prevalence of frailty HR^‡^ 0.53 (0.48 - 0.59) Prevalence of *delirium* HR^‡^ 0.10 (0.07 - 0.14) Risk of *delirium* in frail patients RR^§^ 1.00 (0.51 - 1.98)
Assmann, et al., 2016^26^	Netherlands	Cardiac surgery (TAVI^‡‡^)	To evaluate frailty as an indicator to predict *delirium* and mortality after TAVI^‡‡^.	89	Prospective cohort study	80.4	FI^||^ and Clinical judgment	DSM-4^||||^	Prevalence of frailty HR^‡^ 0.53 (0.43 - 0.63) Prevalence of *delirium* HR^‡^ 0.28 (0.20 - 0.38)
Chew, et al., 2017^27^	Singapore	Surgical	To investigate the association between frailty and incomplete recovery from *delirium* at discharge and to examine the mediating role of incomplete recovery in the relationship between frailty and functional recovery 12 months after *delirium.*	234	Prospective cohort study	84.1±7.1	FI^||^ (20 items) (Frail>0.25)	DRS-R-98^¶¶^	Prevalence of frailty HR^‡^ 0.68 (0.62 - 0.74) Prevalence of *delirium* HR^‡^ 0.23 (0.18 - 0.28) Risk of *delirium* in frail patients RR^§^ 0.90 (0.84 - 0.98)
Ogawa, et al., 2017^28^	Japan	Cardiac surgery	To examine the associations between *delirium* and postoperative frailty and mayor cardiac adverse events.	326	Prospective cohort study	68.6±14.8	Handgrip strength and gait speed	ICDSC^***^	Prevalence of frailty HR^‡^ 0.07 (0.04 - 0.10) Prevalence of *delirium* HR^‡^ 0.13 (0.10 - 0.17) Risk of *delirium* in frail patients RR^§^ 3.16 (1.67 - 5.96)
Gleason, et al., 2017^29^	USA	Orthopedic surgery and Geriatrics service	To stratify frailty in older adults admitted with fractures to determine its association with the postoperative results.	175	Retrospective cohort study	82.3±7.4	FRAIL	Not performed	Prevalence of frailty HR^‡^ 0.42 (0.35 - 0.49) Prevalence of *delirium* HR^‡^ 0.20 (0.15 - 0.27) Risk of *delirium* in frail patients RR^§^ 0.48 (0.26 - 0.87)
Dani, et al., 2018^13^	United Kingdom	Emergency Sector	To evaluate the impact of *delirium* on mortality in a cohort evaluated for frailty.	710	Prospective cohort study	83.1±7.41	FI^||^ (31 items)	CAM^†^	Prevalence of *delirium* HR^‡^ 0.10 (0.08 - 0.13)
Tanaka, et al., 2018^30^	Japan	Liver resection surgery	To apply the Kihon Checklist to evaluate preoperative frailty in older adults to predict outcomes after liver resection.	217	Multicenter and prospective cohort study	75 frail and age 72 non-frail people	KC^†††^ (Frail≥8)	ICDSC^***^	Prevalence of frailty HR^‡^ 0.29 (0.23 - 0.35) Prevalence of *delirium* HR^‡^ 0.12 (0.09 - 0.17) Risk of *delirium* in frail patients RR^§^ 6.52 (1.79 - 23.78)
Giroux, et al., 2018^9^	Canada	Emergency Sector	To evaluate if frailty screening in older adults in the Emergency Department can help identify the risk of *delirium*.	335	Prospective cohort study	76.8±8.1	CFS^‡‡‡^ (Frail>5/7)	CAM^†^	Prevalence of frailty HR^‡^ 0.21 (0.17 - 0.26) Prevalence of *delirium* HR^‡^ 0.12 (0.09 - 0.16) Risk of *delirium* in frail patients RR^§^ 3.79 (2.16 - 6.63)
Alabaf Sabbaghi, et al., 2018^31^	United Kingdom	Emergency Sector	To compare the clinical characteristics, frailty, dementia and *delirium* in a hospital with a specialized counseling team for frail older adults versus usual treatment.	6,191	Retrospective and documentary	84.6±6.3	CFS^‡‡‡^	4-ATM^§§§^	Prevalence of frailty HR^‡^ 0.05 (0.04 - 0.06) Prevalence of *delirium* HR^‡^ 0.12 (0.11 - 0.13)
Nomura, et al., 2018^32^	Japan	Cardiac surgery	To examine the hypothesis that baseline frailty would be associated with postoperative *delirium* and to cognitive changes from 1 to 12 months after a cardiac surgery.	133	Prospective cohort study	Robust 69.3±7.9 Frail 73.4±8.09	Fried’s Phenotype	CAM^†^ and CAM-ICU^||||||^	Prevalence of frailty HR^‡^ 0.34 (0.26 - 0.42) Prevalence of *delirium* HR^‡^ 0.48 (0.40 - 0.56) Risk of *delirium* in frail patients RR^§^ 1.04 (0.68 - 1.58)
Goudzwaard, et al., 2018^33^	Netherlands	Cardiac surgery (TAVI^‡‡^)	To investigate the association between a new Erasmus Frailty Score and short- and long-term results after TAVI^‡‡^.	213	Prospective cohort study	82.0 (IQR: 78.2 - 85.6)	Erasmus Frailty Score (>3/5 of the domains)	Geriatric clinical evaluation	Prevalence of frailty HR^‡^ 0.29 (0.23 - 0.35) Prevalence of *delirium* HR^‡^ 0.20 (0.15 - 0.26)
Geriatric Medicine Research Collaborative, 2019^34^	United Kingdom	Emergency Sector	To evaluate frailty and the patient’s/hospital’s risk factors for *delirium.*	1507	Prospective cohort study	80.0±8.3	CFS^‡‡‡^	4-ATM^§§§^ and DSM-5^¶^	Prevalence of frailty HR^‡^ 0.66 (0.64 - 0.68) Prevalence of *delirium* HR^‡^ 0.15 (0.13 - 0.17) Risk of *delirium* in frail patients RR^§^ 2.83 (2.21 - 3.62)
Saravana-Bawan, et al., 2019^35^	Canada	Emergency general surgery	To evaluate the incidence and risk factors of *delirium* in older adults subjected to emergency surgeries.	332	Prospective cohort study	76.1±7.66	CFS^‡‡‡^	CAM^†^	Prevalence of frailty HR^‡^ 0.24 (0.20 - 0.29) Prevalence of *delirium* HR^‡^ 0.23 (0.19 - 0.28) Risk of *delirium* in frail patients RR^§^ 2.58 (1.76 - 3.79)
Bellelli, et al., 2019^10^	Italy	Acute care (Geriatric emergency)	To evaluate if frailty is associated with *delirium* and if it affects performance in three attention tests.	89	Prospective cohort study	83.15±6.05	FI^||^ (38 items)	4-ATM^§§§^ and DSM-5^¶^	Prevalence of frailty HR^‡^ 0.47 (0.37 - 0.58) Prevalence of *delirium* HR^‡^ 0.42 (0.32 - 0.52) Risk of *delirium* in frail patients RR^§^ 1.84 (1.10 - 3.09)
Goudzaard, et al., 2020^36^	Netherlands	Cardiac surgery (TAVI^‡‡^)	To investigate the incidence, the determinants and the consequences of postoperative *delirium* in aged patients subjected to TAVI^‡‡^.	543	Prospective cohort study	79.1±8.0	Erasmus Frailty Score (>3/5 of the domains)	Geriatric clinical evaluation	Prevalence of frailty HR^‡^ 0.28 (0.25 - 0.32) Prevalence of *delirium* HR^‡^ 0.14 (0.11 - 0.17) Risk of *delirium* in frail patients RR^§^ 2.22 (1.44 - 3.42)
Itagaki, et al., 2020^37^	Japan	Cardiac surgery	To examine how physical frailty and cognitive impairment affect the incidence of *delirium* after cardiac surgeries in older patients.	89	Retrospective study	74.9±5.5	j-CHS^¶¶¶^	ICDSC^***^	Prevalence of frailty HR^‡^ 0.27 (0.19 - 0.37) Prevalence of *delirium* HR^‡^ 0.35 (0.26 - 0.45) Risk of *delirium* in frail patients RR^§^ 2.51 (1.24 - 5.08)
Saljuqi, et al., 2020^38^	USA	Emergency general surgery	To evaluate the impact of frailty on *delirium* and on other outcomes in geriatric patients subjected to emergency general surgeries.	163	Prospective cohort study	71±7	EGSSFI^****^	CAM^†^	Prevalence of *delirium* HR^‡^ 0.26 (0.20 - 0.33) Risk of *delirium* in frail patients RR^§^ 2.50 (1.50 - 4.17)
Susano, et al., 2020^39^	USA	Orthopedic surgery (spine)	To test the hypothesis that preoperative screening for frailty or cognitive impairment identifies patients at risk for postoperative *delirium* (primary endpoint).	229	Prospective cohort study	≥70 to 75	FRAIL	CAM^†^	Prevalence of frailty HR^‡^ 0.24 (0.19 - 0.30) Prevalence of *delirium* RR^§^ 0.25 (0.20 - 0.31)
Mahanna-Gabrielli, et al., 2020^40^	USA	Major non-cardiac surgery	To examine the relationship between frailty and postoperative *delírium* after a large-size non-cardiac surgery.	167	Prospective cohort study	70	FRAIL	CAM-ICU^||||||^	Prevalence of frailty HR^‡^ 0.19 (0.13 - 0.25) Prevalence of *delirium* HR^‡^ 0.25 (0.19 - 0.32) Risk of *delirium* in frail patients RR^§^ 1.78 (1.01 - 3.13)
Chen; Qin, 2021^41^	China	Orthopedic surgery	To examine the discriminatory value of the Frailty Index to predict postoperative *delirium* and cognitive dysfunction after total hip arthroplasty.	383	Prospective cohort study	72 (from 65 to 85)	FI^||^ (11 items) Frail>0.18)	DSM-5^¶^	Prevalence of frailty HR^‡^ 0.54 (0.49 - 0.59) Prevalence of *delirium* HR^‡^ 0.17 (0.14 - 0.21) Risk of *delirium* in frail patients RR^§^ 1.70 (1.06 - 2.72)

*CI = Confidence Interval; ^†^CAM = Confusion Assessment Method; ^‡^HR = Hazard Ratio; §RR = Relative Risk; ^|^FI = Frailty Index; ^¶^DSM-5 = Diagnostic and Statistical Manual of Mental Disorders - Fifth edition; ^**^CHS = Cardiovascular Health Study; ^††^SOF = Study of Osteoporotic Fracture; ^‡‡^TAVI = Transcatheter Aortic Valve Implantation; ^§§^EFS = Edmonton Frail Scale; ^||||^DSM-4 = Diagnostic and Statistical Manual of Mental Disorders *-* Fourth edition; ^¶¶^DRS-R-98 = *Delirium* Rating Scale-Revised-98; ^***^ICDSC = Intensive Care Delirium Screening Checklist; ^†††^KC = Kihon Checklist; ‡‡‡CFS = Clinical Frailty Scale; ^§§§^4-ATM = Abbreviated Mental Test; ^||||||^CAM-ICU = Confusion Assessment Method for the Intensive Care Unit; ^¶¶¶^j-CHS = Japanese version of the Cardiovascular Health Study; ^****^EGSSFI = Emergency General Surgery Specific Frailty Index

**Figure 4:**
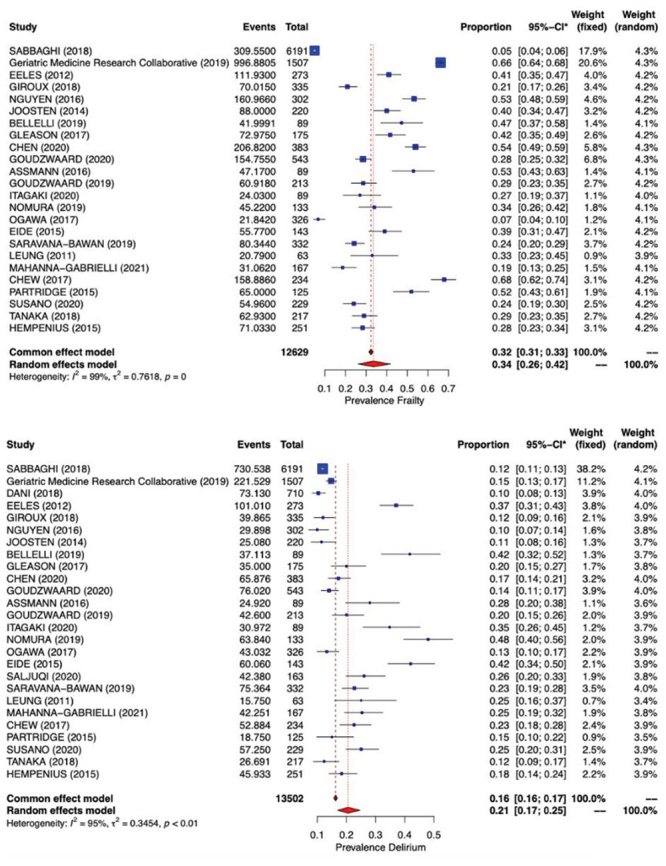
Estimated prevalence of physical frailty and *delirium* in the meta-analysis model. Curitiba, PR, Brazil, 2021

Note: Heterogeneity was tested by means of the I^2^ test, considering it significant when *p*<0.05. *CI = Confidence Interval

In Figure 4, it can be seen that the prevalence of frailty in the combined effect of all studies was 34% (from 26% to 42%) *I* ^2^=99%; *t* ^2^=0.7618, *p*=0, and that the value for *delirium* was 21% (from 17% to 25%), with I^2^=95%; t^2^=0.3454, *p*<0.01.

**Figure 5:**
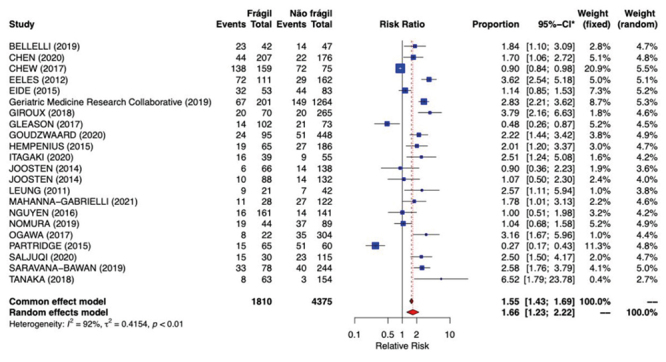
Estimated relative risk for *delirium* among the frail patients in the meta-analysis model. Curitiba, PR, Brazil, 2021

Note: Heterogeneity was tested by means of the I^2^ test, considering it significant when *p*<0.05. *CI = Confidence Interval

In Figure 5, the relative risk of frailty and delirium was 1.66 (from 1.18 to 2.22; I^2^=92%; t^2^=0.4154, p<0.01). Each line represents a study, and the last one represents the combination of the results (meta-analysis), which is symbolized by a “diamond”. 

## Discussion

In this systematic review with meta-analysis, it was identified that frailty was independently associated with an increased risk of *delirium* in hospitalized older adults: 1.66 (95% CI: 1.23-2.22 I^2^=95%; t^2^=0.4154, *p*<0.01). The prevalence of frailty in hospitalized older adults was 34% (from 26% to 42%) and that of *delirium* across the studies was 21% (from 17% to 25%).

The scarcity of studies evaluating frailty as a predisposing factor to *delirium* ^10^ ^,^ ^21^ ^,^ ^37^ ^-^ ^38^ ^,^ ^41^ was an unexpected finding of this paper, as the association between these conditions is accepted in the clinical practice. No relationship was observed between frailty and development of *delirium* in seven of the 26 studies analyzed, (RR: 1; 95% CI: 0.51-1.98)^25^, (RR: 0.90; 95% CI: 0.36-2.23)^21^, (RR: 0.48; 95% CI: 0.26-0.87)^29^, (RR: 1.04; 95% CI: 0.68-1.54)^32^, (RR: 1.14; 95% CI: 0.85-1.53)^23^, (RR: 0.90; 95% CI: 0.84-0.98)^27^, (RR: 0.27; 95% CI: 0.17-0.43)^24^.

The instruments used to evaluate frailty showed great heterogeneity, with preference given to the use of multidimensional instruments. Evaluation by Fried’s frailty phenotype^32^ was used in only one study; however, its markers were used in other studies, which worked with Fried’s modified phenotype^19^, or some of its components (gait speed and handgrip strength)^28^. Only one of the studies used a frailty index associated with clinical judgment^26^. 

*Delirium* was evaluated using validated diagnostic instruments and screening tools, with significant heterogeneity across the studies. The screening instrument most frequently used was the Confusion Assessment Method (CAM)^9^ ^,^ ^13^ ^,^ ^19^ ^,^ ^21^ ^-^ ^23^ ^,^ ^35^ ^,^ ^38^. Other studies have used the association of CAM with other instruments, such as CAM-ICU^32^ and/or DSM diagnostic criteria^38^. Used in critically-ill patients, CAM-ICU was also used separately^39^.

4-AT, a faster evaluation instrument, was used separately^31^ or associated with the DMS-V criteria^10^ ^,^ ^34^. The diagnostic criteria were used alone, DMS-IV^25^, and/or associated with other POD/DMS-V^41^. Other ways of detecting *delirium* were employed, such as geriatric evaluation^33^ ^,^ ^36^ and ICDSC^28^ ^,^ ^30^ ^,^ ^37^. Some studies did not specify the detection method^24^ ^-^ ^25^ ^,^ ^29^.

The prevalence of frailty in hospitalized older adults was 34% (from 23% to 46%). The highest prevalence values of frailty were observed in a study conducted in Singapore with 234 older adults with surgical indication in which the association between frailty and residual subsyndromic *delirium* was investigated: 68% (from 62% to 74%)^27^. The prospective multicenter study conducted in 45 hospitals from the United Kingdom with a sample of 1,507 patients also reached high percentages of frailty and values close to the study developed in Singapore: 66% (from 64% to 68%)^34^.

A number of studies developed in China and Australia have found slightly lower percentages of frailty when compared to the aforementioned studies. In China, with a sample consisting of 383 older adults, the study aimed at examining the MFI discriminatory value to predict *delirium* and cognitive dysfunction after hip arthroplasty, and 54% (from 62% to 74%) prevalence was observed^40^. In Australia, the study developed with 302 aged patients hospitalized with atrial fibrillation showed 53% (from 48% to 59%) prevalence^25^.

The prevalence of *delirium* among the studies was 21% (from 17% to 24%). The highest prevalence observed was found in the study conducted in Japan, 48% (95% CI: 40%-56%), with 133 patients that evaluated the association of baseline frailty with postoperative *delirium* and cognitive change 1 and 12 months after cardiac surgeries^32^. In the prospective cohort study conducted in Italy with 89 older adults, evaluating frailty and *delirium* in patients admitted to a geriatric emergency service, the prevalence of *delirium* was 42% (from 32% to 52%)^10^.

The mechanisms surrounding development of *delirium* in frail patients are complex: these patients experience decreased functional capacity and increased vulnerability when subjected to a stressor, such as major surgery or an acute critical medical situation, making it more likely that they will experience *delirium.* Frail older adults also have cognitive impairment, which intensifies the risk of *delirium* ^12^. 

From a clinical point of view, frailty can be considered a risk factor for development of *delirium*, although there is still not sufficient evidence that *delirium* can be a trigger for frailty. When persistent, *delirium* can be a precipitating factor for deterioration in terms of frailty. In the evaluation of the hospitalized older adults, frailty should be screened for, as it allows anticipating occurrence of *delirium*. Likewise, systematic screening for *delirium* should be performed to identify individuals at risk for subsequent deterioration in terms of frailty^43^.

Active search for the frailty condition in the acute care setting (hospitalized patients) is mandatory, and an individualized approach is required in the management of frail older adults^44^, due to the higher association with hospital complications^45^. 

A cohort study conducted with 710 older adults in a hospital with patients over 70 years old evidenced that both *delirium* and frailty independently increase the risk of death, *delirium* (HR: 2.4; 95% CI: 1.8-3.3, p<0.01) and frailty (HR 3.5, 95% CI: 1.2-9.9, p=0.02). The risk of death is higher in patients with *delirium* at all frailty levels, which highlights the importance of preventing, detecting and treating *delirium* in any patient and recognizing it as a serious condition that interferes with prognosis^13^. 

Frailty is a dynamic entity, and older adults can transition from being robust to being frail^46^. Little is said about the specific approach to physical frailty in the hospitalized patient, with its relationship to morbidities, mortality, and/or *delirium* being more evaluated. A comprehensive care plan for frailty should systematically address the following: polypharmacy, management of sarcopenia, treatable causes of weight loss and causes of exhaustion (depression, anemia, hypotension, hypothyroidism, and vitamin B12 deficiency), with strong recommendation, although with too low certainty of evidence^47^.

Although more studies are needed to better clarify the cause/effect relationship between these two conditions, this association has important clinical implications. The presence of frailty should be investigated in hospitalized aged patients, as this condition predicts negative adverse outcomes and requires individualized care. When present, frailty should lead to a search for the presence of concomitant *delirium*, given the high probability of its simultaneous incidence. In the absence of *delirium*, evidence-based non-pharmacological measures should be intensively implemented to prevent it^48^, given the high risk for its development. 

Programs involving multiple components conducted by different professionals in the prevention of *delirium* have the potential to reduce complications in high-risk aged patients, thereby improving treatment and long-term quality of life. The implementation of additional interprofessional teams acting to prevent *delirium* and providing regular training on the optimal management of *delirium* is an intervention option. Demonstrating the effectiveness of these programs requires large multicenter studies^49^. 

The methodological quality of the studies was evaluated as reasonable to good (not excellent) and they were heterogeneous with regard to study populations and definitions of the variables of interest (frailty and *delirium*). The quality levels of the studies evaluated did not influence the association between frailty and subsequent *delirium*, but the risk of bias was relevant because many studies did not adjust for confounding factors. Most of the studies included in the current systematic review evidenced the association between the frailty and *delirium* variables, and it is up to health professionals to evaluate older adults with adequate instruments to detect the frailty syndrome associated with development of *delirium*.

The strengths of this study include the comprehensive search strategy, the methodological evaluation, and the standardized data extraction process. The limitations of this systematic review are the heterogeneous and specific populations of the studies included, the sample sizes (not always representative of the population), and the different methods to evaluate frailty and *delirium*. 

The dichotomous evaluation of frailty and *delirium* may be another bias, as these conditions can be classifiable in terms of severity. Another possible limitation relates to the way of evaluating *delirium*, which was assessed only once, daily and or every other day, not considering the possibility that the condition may fluctuate throughout the day; therefore, it is possible that *delirium* was undersampled in some studies.

Identification of baseline frailty raises the possibility that it may be a potential therapeutic target in the prevention of *delirium* in the clinical practice. The results of this review may assist in encouraging early diagnosis of the frailty syndrome and *delirium* in the hospital setting, guiding prognosis, individualized care plans, and prevention of adverse outcomes.

Efforts should be directed towards mitigation and treatment strategies of *delirium* with early identification of risk factors^50^, in different clinical and surgical contexts. 

Studies of the association between frailty and *delirium* in hospitalized older adults are still incipient, which highlights the need to investigate interventions for hospitalized older adults with frailty and *delirium*.

## Conclusion

This study showed 34% prevalence of frailty and 21% of *delirium* in hospitalized older adults, with frailty being an independent risk factor for developing *delirium*, with an increased chance of 66% when compared to non-frail individuals.
